# Correction: Task-Based Core-Periphery Organization of Human Brain Dynamics

**DOI:** 10.1371/journal.pcbi.1003617

**Published:** 2014-04-11

**Authors:** The PLOS Computational Biology Staff

The table legends in the files Table S1, Table S2 and Table S3 are incorrectly titled. The correct titles are given below:

Table S1. Experimental details for behavioral data acquired between scanning sessions.

Table S2. Experimental details for brain imaging data acquired during scanning sessions.

Table S3. Brain regions in the Harvard-Oxford (HO) cortical and subcortical parcellation scheme provided by FSL [83], [84].

The table legend in the file for Table S3 is also missing two references. These are number 83 and 84 from the main reference list.

The corrected files can be viewed below.

## Supporting Information

Table S1 **Experimental details for behavioral data acquired between scanning sessions.** We give the minimum, mean, maximum, and standard error of the mean over participants for the following variables: the number of days between scanning sessions; the number of practice sessions performed at home between scanning sessions; and the number of trials composed of extensively, moderately, and minimally trained sequences during home practice between scanning sessions.

Table S2 **Experimental details for brain imaging data acquired during scanning sessions.** In the top three rows, we give the mean, minimum, maximum, and standard error over participants for the number of blocks composed of extensively, moderately, and minimally trained sequences during scanning sessions. In the bottom three rows, we give (in TRs) the mean, minimum, maximum, and standard error of the length over blocks composed of extensively, moderately, and minimally trained sequences during scanning sessions.

Table S3 **Brain regions in the Harvard-Oxford (HO) cortical and subcortical parcellation scheme provided by FSL [83], [84]** and their affiliation to the temporal core (C; cyan), bulk (B; gold), and periphery (P; maroon) for both left (L) and right (R) hemispheres.
