# Supplementary material for: Task-Based Core-Periphery Organization of Human Brain Dynamics
Source: PLoS Comput Biol. 2013 Sep 26;9(9):e1003171. doi: 10.1371/journal.pcbi.1003171 (PMC3784512; doi:10.1371/journal.pcbi.1003171)
Supplement: Table S1 — Experimental details for behavioral data acquired between scanning sessions. We give the minimum, mean, maximum, and standard error of the mean over participants for the following variables: the number of days between scanning sessions; the number of practice sessions performed at home between scanning sessions; and the number of trials composed of extensively, moderately, and minimally trained sequences during home practice between scanning sessions. (PDF) [file pcbi.1003171.s009.pdf]

	Mean	Minimum	Maximum	Standard Error
Days				
Between Scans 1 and 2	12.00	9	14	0.34
Between Scans 2 and 3	12.45	10	14	0.29
Between Scans 3 and 4	12.10	9	22	0.63
Practice Sessions				
Between Scans 1 and 2	9.70	8	10	0.14
Between Scans 2 and 3	9.75	4	14	0.44
Between Scans 3 and 4	10.05	7	13	0.32
Extensively Trained Trials				
Between Scans 1 and 2	620.80	512	640	9.40
Between Scans 2 and 3	624.00	256	896	28.57
Between Scans 3 and 4	643.20	448	832	20.48
Moderately Trained Trials				
Between Scans 1 and 2	97.00	80	100	1.46
Between Scans 2 and 3	97.50	40	140	4.46
Between Scans 3 and 4	100.50	70	130	3.20
Minimally Trained Trials				
Between Scans 1 and 2	9.70	8	10	0.14
Between Scans 2 and 3	9.75	4	14	0.44
Between Scans 3 and 4	10.05	7	13	0.32

Table 1: **Experimental Details for Behavioral Data Acquired Between Scanning Sessions.** We give the minimum, mean, maximum, and standard error of the mean over participants for the following variables: the number of days between scanning sessions; the number of practice sessions performed at home between scanning sessions; and the number of trials composed of extensively, moderately, and minimally trained sequences during home practice between scanning sessions.
