# Supplementary material for: Task-Based Core-Periphery Organization of Human Brain Dynamics
Source: PLoS Comput Biol. 2013 Sep 26;9(9):e1003171. doi: 10.1371/journal.pcbi.1003171 (PMC3784512; doi:10.1371/journal.pcbi.1003171)
Supplement: Table S2 — Experimental details for brain imaging data acquired during scanning sessions. In the top three rows, we give the mean, minimum, maximum, and standard error over participants for the number of blocks composed of extensively, moderately, and minimally trained sequences during scanning sessions. In the bottom three rows, we give (in TRs) the mean, minimum, maximum, and standard error of the length over blocks composed of extensively, moderately, and minimally trained sequences during scanning sessions. (PDF) [file pcbi.1003171.s010.pdf]

	Minimum	Mean	Maximum	Standard Error
Extensively Trained Blocks				
During Scan 1	6.00	9.70	10.00	0.21
During Scan 2	10.00	10.00	10.00	0.00
During Scan 3	10.00	10.00	10.00	0.00
During Scan 4	8.00	9.90	10.00	0.10
Moderately Trained Blocks				
During Scan 1	5.00	9.70	11.00	0.27
During Scan 2	10.00	10.00	10.00	0.00
During Scan 3	10.00	10.00	10.00	0.00
During Scan 4	8.00	9.90	10.00	0.10
Minimally Trained Blocks				
During Scan 1	7.00	9.80	11.00	0.18
During Scan 2	10.00	10.00	10.00	0.00
During Scan 3	10.00	10.00	10.00	0.00
During Scan 4	8.00	9.90	10.00	0.10
Length of Extensively Trained Blocks				
During Scan 1	52.50	61.94	72.20	1.34
During Scan 2	35.50	42.36	45.90	0.72
During Scan 3	35.40	40.79	45.50	0.77
During Scan 4	34.60	40.30	45.70	0.87
Length of Moderately Trained Blocks				
During Scan 1	50.80	61.67	72.60	1.26
During Scan 2	39.70	47.56	57.20	0.80
During Scan 3	37.60	45.07	52.80	0.67
During Scan 4	37.60	43.83	50.60	0.79
Length of Minimally Trained Blocks				
During Scan 1	52.10	61.19	70.60	1.29
During Scan 2	44.10	50.02	57.70	0.73
During Scan 3	42.50	47.37	54.50	0.71
During Scan 4	39.70	45.79	54.10	0.70

Table 1: **Experimental Details for Brain Imaging Data Acquired During Scanning Sessions.** In the top three rows, we give the mean, minimum, maximum, and standard error over participants for the number of blocks composed of extensively, moderately, and minimally trained sequences during scanning sessions. In the bottom three rows, we give (in TRs) the mean, minimum, maximum, and standard error of the length over blocks composed of extensively, moderately, and minimally trained sequences during scanning sessions.
