# Supplementary material for: Task-Based Core-Periphery Organization of Human Brain Dynamics
Source: PLoS Comput Biol. 2013 Sep 26;9(9):e1003171. doi: 10.1371/journal.pcbi.1003171 (PMC3784512; doi:10.1371/journal.pcbi.1003171)
Supplement: Table S3 — Brain regions in the Harvard-Oxford (HO) cortical and subcortical parcellation scheme provided by FSL [83], [84] and their affiliation to the temporal core (C; cyan), bulk (B; gold), and periphery (P; maroon) for both left (L) and right (R) hemispheres. (PDF) [file pcbi.1003171.s011.pdf]

Region Name	Affiliation		Region Name	Affiliation	
Frontal pole	B(R)	B(L)	Cingulate gyrus, anterior	B(R)	B(L)
Insular cortex	B(R)	B(L)	Cingulate gyrus, posterior	P(R)	B(L)
Superior frontal gyrus	B(R)	B(L)	Precuneus cortex	B(R)	B(L)
Middle frontal gyrus	B(R)	B(L)	Cuneus cortex	C(R)	C(L)
Inferior frontal gyrus, pars triangularis	B(R)	P(L)	Orbital frontal cortex	B(R)	B(L)
Inferior frontal gyrus, pars opercularis	B(R)	B(L)	Parahippocampal gyrus, anterior	B(R)	B(L)
Precentral gyrus	C(R)	C(L)	Parahippocampal gyrus, posterior	B(R)	P(L)
Temporal pole	B(R)	B(L)	Lingual gyrus	C(R)	C(L)
Superior temporal gyrus, anterior	B(R)	B(L)	Temporal fusiform cortex, anterior	P(R)	B(L)
Superior temporal gyrus, posterior	B(R)	B(L)	Temporal fusiform cortex, posterior	P(R)	P(L)
Middle temporal gyrus, anterior	B(R)	B(L)	Temporal occipital fusiform cortex	P(R)	P(L)
Middle temporal gyrus, posterior	B(R)	B(L)	Occipital fusiform gyrus	P(R)	P(L)
Middle temporal gyrus, temporooccipital	P(R)	P(L)	Frontal operculum cortex	B(R)	P(L)
Inferior temporal gyrus, anterior	B(R)	B(L)	Central opercular cortex	B(R)	B(L)
Inferior temporal gyrus, posterior	B(R)	B(L)	Parietal operculum cortex	P(R)	B(L)
Inferior temporal gyrus, temporooccipital	B(R)	B(L)	Planum polare	C(R)	B(L)
Postcentral gyrus	P(R)	C(L)	Heschl's gyrus	C(R)	C(L)
Superior parietal lobule	B(R)	C(L)	Planum temporale	B(R)	B(L)
Supramarginal gyrus, anterior	B(R)	C(L)	Supercalcarine cortex	C(R)	C(L)
Supramarginal gyrus, posterior	B(R)	P(L)	Occipital pole	C(R)	B(L)
Angular gyrus	P(R)	B(L)	Caudate	P(R)	B(L)
Lateral occipital cortex, superior	P(R)	P(L)	Putamen	P(R)	B(L)
Lateral occipital cortex, inferior	P(R)	B(L)	Globus pallidus	P(R)	B(L)
Intracalcarine cortex	C(R)	C(L)	Thalamus	P(R)	P(L)
Frontal medial cortex	B(R)	B(L)	Nucleus Accumbens	B(R)	B(L)
Supplemental motor area	C(R)	C(L)	Parahippocampal gyrus	B(R)	B(L)
Subcallosal cortex	B(R)	B(L)	Hippocampus	B(R)	B(L)
Paracingulate gyrus	B(R)	B(L)	Brainstem	B(R)	B(L)

Table 1: **Brain regions in the Harvard-Oxford (HO) Cortical and Subcortical Parcellation Scheme provided by FSL** [?, ?] and their affiliation to the temporal core (C; cyan), bulk (B; gold), and periphery (P; maroon) for both left (L) and right (R) hemispheres.
