# Supplementary material for: Task-Based Core-Periphery Organization of Human Brain Dynamics
Source: PLoS Comput Biol. 2013 Sep 26;9(9):e1003171. doi: 10.1371/journal.pcbi.1003171 (PMC3784512; doi:10.1371/journal.pcbi.1003171)
Supplement: Text S1 — Supplementary materials for “Task-Based Core-Periphery Organization of Human Brain Dynamics”. In this document, we provide additional results to demonstrate the reliability of temporal core-periphery organization, the reliability of geometrical core-periphery organization, and the relationship between temporal core-periphery organization and community structure. We also include a length section on important methodological considerations. (PDF) [file pcbi.1003171.s012.pdf]

Text S1: Supplementary Materials for “Task-Based Core-Periphery Organization of Human Brain Dynamics”

Danielle S. Bassett[1,2,*], Nicholas F. Wymbs[5], M. Puck Rombach[3,4], Mason A. Porter[3,4], Peter J. Mucha[6,7], Scott T. Grafton[5]

[1]Department of Physics, University of California, Santa Barbara, CA 93106, USA;

[2] Sage Center for the Study of the Mind, University of California, Santa Barbara, CA 93106;

[3] Oxford Centre for Industrial and Applied Mathematics, Mathematical Institute, University of Oxford, Oxford OX1 3LB, UK;

[4]CABDyN Complexity Centre, University of Oxford, Oxford, OX1 1HP, UK;

[5]Department of Psychological and Brain Sciences and UCSB Brain Imaging Center, University of California, Santa Barbara, CA 93106, USA;

[6]Carolina Center for Interdisciplinary Applied Mathematics, Department of Mathematics, University of North Carolina, Chapel Hill, NC 27599, USA;

[7]Institute for Advanced Materials, Nanoscience & Technology, University of North Carolina, Chapel Hill, NC 27599, USA;

[*]Corresponding author. Email address: `dbassett@physics.ucsb.edu`

July 29, 2013

Reliability of Temporal Core-Periphery Organization

A brain region's role in the temporal core, bulk, and periphery is robust across levels of training. Regions identified as part of the core, bulk, or periphery in multilayer networks constructed from the EXT blocks in scanning session 1 have similar flexibilities in the other two levels of training (MOD and MIN; see Fig. S1A) for the same scanning session. To quantify the variability of a brain region's flexibility, we calculated the coefficient of variation (CV) of flexibility over the 100 optimizations and the 3 levels of training (see Fig. S1B). The CV is defined as $\text{CV} = \sigma/\mu$, where σ is the standard deviation of a given sample and μ is its mean. We observe that the variabilities over optimizations and scans (i.e., CV) and over participants (i.e., error bars) are largest in regions designated as part of the temporal core and smallest in regions designated as part of the temporal periphery.

In addition, regional flexibility is also conserved across both intensity of training (MIN, MOD, and EXT) and duration of training (sessions 1–4). Observe in Fig. S2 that regions identified as part of the temporal core in multilayer networks constructed from the EXT blocks in scanning session 1 exhibit small flexibility for all other scanning sessions and for all 3 training levels (EXT, MOD, and MIN). Regions in the temporal bulk and temporal periphery exhibit a similar amount of flexibility to one another.

Reliability of Geometrical Core-Periphery Organization

As we illustrate in Fig. S3, the geometrical core-periphery organization of the brain was consistent over the 42 days of practice, across sequence types, and throughout variations in the intensity of training (MIN, MOD, and EXT) and in the duration of training (sessions 1–4).

Relationship Between Temporal Core-Periphery Organization and Community Structure

The division of the brain networks into temporal core, bulk, and peripheral nodes has interesting similarities to their partitioning into communities based on optimizing multilayer modularity. We first noted this similarity when we examined community structure in an object that we call the *mean-coherence matrix.* The mean-coherence matrix $\bar{\mathbf{A}}$ contain elements $\bar{A}_{ij}$ that are equal to the mean coherence between nodes i and j over participants and EXT blocks on day 1 of the experiment. We determined the community structure of this mean-coherence matrix by optimizing the single-layer modularity quality function [1, 2, 3, 4, 5]:

$$Q_{\text{single-layer}} = \sum_{ij} \left[\bar{A}_{ij} - \frac{k_i k_j}{2m} \right] \delta(g_i, g_j) \,, \tag{1}$$

where node i is assigned to community g_i, node j is assigned to community g_j, the Kronecker delta $\delta(g_i, g_j) = 1$ if $g_i = g_j$ and it equals 0 otherwise, k_i is the strength of node i, and m is the mean strength of all nodes in the network. After optimizing this single-layer quality function 100 times, we constructed a representative partition [6] from the set of 100 partitions. (Each partition arises from a single optimization.) One community in this representative partition, which we show in Fig. S4A, appears to have high connectivity to the other two communities: nodes in this first community have edges with strong weights to nodes in the other two communities. This indicates a high coherence in the BOLD time series, and this behavior is consistent with the behavior expected from a network "core". A second community in this representative partition appears to have low connectivity to the other two communities: nodes in this community have edges with small weights that connect to nodes in the other two communities. This indicates a low coherence in the BOLD time series, and this behavior is consistent with the behavior expected from a "periphery".

It is important to note that we observed this relationship between temporal core-periphery organization and community structure in networks encoded by *mean matrices*. However, networks encoded by *mean matrices* constructed by averaging correlation-based matrices often do not adequately represent the topological or geometrical structure of the ensemble of individual networks from which they are derived [7]. We therefore test for a relationship between the temporal core-periphery organization and community structure in the ensemble of networks extracted from individual participants.

A division of the brain into temporal core, bulk, and peripheral regions gives a partition of the functional brain network. We label this partition using the Greek letter ν, and we use the z-score of the Rand coefficient [8] to test for similarities between this partition and algorithmic partitions, which we label using η, into

communities (based on optimization of multilayer modularity) for each participant, block, and optimization. For each pair of partitions ν and η, we calculate the Rand z-score in terms of the total number of node pairs M in the network, the number of pairs M_ν that are in the same community in partition ν but not in the partition η, the number of pairs M_η that are in the same community in partition η but not in ν, and the number of node pairs $w_{\nu\eta}$ that are assigned to the same community in both partition ν and partition η. The z-score of the Rand coefficient allows one to compare partitions η and ν, and it is given by the formula

$$z_{\nu\eta} = \frac{1}{\sigma_{w_{\nu\eta}}} w_{\nu\eta} - \frac{M_\nu M_\eta}{M}\,, \tag{2}$$

where $\sigma_{w_{\nu\eta}}$ is the standard deviation of $w_{\nu\eta}$. Let the *mean partition similarity* z^i denote the mean value of $z_{\nu\eta}$ over all partitions η (i.e., for all blocks and all optimizations) for participant i.

As we show in Fig. S4B-D, we find that communities identified by the optimization of the multilayer modularity quality function (see the "Materials and Methods" section in the main manuscript) have significant overlap with the division into temporal core, bulk, and periphery during early learning. The mean values of z^i over participants indicate that there is a significant similarity between the partitions into modules and the partitions into core, bulk, and periphery for networks representing functional connectivity during blocks of extensively, moderately, and minimally trained sequences on scanning day 1. This similarity between community structure and temporal core-periphery organization is also evident for blocks of moderately and minimally trained sequences practiced during later scanning sessions. These results underscore the fact that core-periphery organization can be consistent with community structure. Note, however, that there is no statistical similarity between partitions into core, bulk, and periphery and partitions into communities for later learning. (As shown in Fig. S4B-D, the z-scores for networks that represent the functional connectivity during extensive training in scans 2–4, moderate training in scans 3–4, and minimal training in scan 4 are not significantly greater than expected (i.e., under the null hypothesis of no difference between the partitions).) Together, this set of results suggests that the relationship between these two types of mesoscale organization can be altered by learning.

Methodological Considerations

Experimental Factors

Effect of Region Size

Recent studies have noted that brain-region size can affect estimates of hard-wired connectivity strength used in constructing structural connectomes [9, 10]. Although the present work is concerned with functional connectomes, it is nevertheless relevant to consider whether or not region size could be a driving effect of the observed core-periphery organization. Importantly, we observe no significant correlation between region size and flexibility (see Fig. S5), which suggests that region size is not driving the reported results.

Effect of Block Design

Another important factor is the underlying experimental block design and its effect on the correlation structure between brain regions in a single time window (i.e., in a single layer in the multilayer formalism). Two brain regions, such as motor cortex (M1) and supplementary motor area (SMA), might be active during the trial but quiet during the inter-trial interval (ITI). This would lead to a characteristic on-off activity pattern that is highly correlated with all other regions that also turn on with the task and off during the ITI. The frequency of this task-related activity (one on-off cycle per trial, where each trial is of length 4–6 TRs) is included in our frequency band of interest (wavelet scale two, whose frequency range is 0.06–0.12 Hz), and it therefore likely plays a role in the observed correlation patterns between brain regions in a single time window.

Note, however, that our investigations of dynamic network structure—namely, our computations of flexibility of community allegiance—probe functional connectivity dynamics at much larger time scales, and the associated frequencies are an order of magnitude smaller. They lie in the range 0.0083–0.012 Hz, as there is one time window every 40–60 TRs. At these longer time scales, we can probe the effects of both early learning and extended learning independently of block-design effects.

Specificity of Dynamic Network Organization as a Predictor of Learning

An important consideration is whether there exist (arguably) simpler properties of brain function than flexibility that could be used to predict learning. We find that the power of activity, the mean connectivity strength, and parameter estimates from a general linear model (GLM) provide less predictive power than flexibility.

Measures of Activity and Connectivity. It is far beyond the scope of this study to perform exhaustive computations using all possible measures of brain-region activity, so we focus on two common diagnostics. One is based on functional connectivity, and the other is based on brain activity. To estimate the strength of functional connectivity, we calculated the mean pairwise coherence between regional wavelet scale-two time series constructed from the BOLD signal, where we took the mean over all possible pairs of regions and all EXT experimental blocks extracted from scans on day 1 for a given subject. To estimate the strength of activity, we calculated the mean signal power of the regional wavelet scale-two time series constructed from the BOLD signal, where we took the mean over all regions and all EXT experimental blocks extracted from scans on day 1 for a given subject. We estimate the power P_{w_2} of the wavelet scale-two time series as the square of the time series normalized by its length:

$$P_{w_2} = \sum_t \frac{w_2(t)^2}{T}\,, \tag{3}$$

where T is the length of the time series [11, 12].

We found that neither mean pairwise coherence nor mean power of regional activity measured during the first scanning session could be used to predict learning during the subsequent 10 home training sessions. For the mean pairwise coherence, we obtained a Pearson correlation of $r \doteq -0.003$ and a p-value of $p \doteq 0.987$. For the mean power of brain-region activity, we obtained $r \doteq -0.218$ and $p \doteq 0.354$. These results indicate that a prediction similar to that made using the flexibility is not possible using the (arguably) simpler properties of the mean pairwise coherence or the mean power of regional brain activity. They also suggest that the dynamic pattern of coherent functional brain activity is more predictive than means of such activity patterns.

Parameter Estimates for a General Linear Model. We determined relative differences in the BOLD signal by using a GLM approach for event-related functional data [13, 14]. For each participant, we constructed a single design matrix for event-related fMRI by specifying the onset time and duration of all stimulus events from each scanning session (i.e., the pre-training session and the 3 test sessions). We found estimations of changes in the BOLD signal related to experimental conditions by using the design matrix with the GLM. We modeled the duration of each sequence trial as the time elapsed to produce the entire sequence; in other words, we calculated the movement time (MT), which is a direct measure of the time spent on a task and leads to accurate modeling of BOLD signals using the GLM [15]. Separate stimulus vectors indicate each sequence exposure type (EXT, MOD, and MIN) for each scanning session. We took potential differences in brain activity due to rate of movement into account by using the MT for each trial as the modeled duration for the corresponding event. We convolved events using the canonical hemodynamic response function and temporal derivative. Using the canonical hemodynamic response function (HRF) and its temporal derivative — we use the implementation in the Statistical Parametric Mapping Toobox (SPM8) [18] — we then modeled the events that were specified in the stimulus vectors. From this procedure, we obtained a pair of beta images for each event type. These images correspond to estimates of the HRF and its temporal derivative. Using freely available software [16], we then combined the corresponding beta image pairs for each event type (HRF and its temporal derivative) at the voxel level to form a magnitude image [17]

$$H = \mathrm{sign}(\hat{B}_1) + \sqrt{(\hat{B}_1 + \hat{B}_2)}\,, \tag{4}$$

where H is called the "combined amplitude" of the estimation of the BOLD signal using the HRF ($\hat{B}_1$) and its temporal derivative ($\hat{B}_2$). [1] This yielded separate magnitude images for each sequence exposure type

[1] In this equation, we use the hat notation to indicate that these values are estimated (rather than directly measured) from a general linear model for a response variable (such as regional cerebral blood) at each voxel in a given participant [14].

(EXT, MOD, and MIN) and session. We then calculated the mean region-based magnitude for each exposure type and session using regions derived from each subject's grey matter-constrained Harvard-Oxford (HO) atlas.

We did not find a significant correlation between the mean parameter estimates averaged over brain regions for the EXT trials in scanning session 1 and learning of the EXT sequences over the subsequent approximately 10 home training sessions. The Pearson correlation is $r \doteq -0.10$ and the p-value is $p \doteq 0.65$.

Subject State-Dependence of Dynamic Network Organization

Our finding that temporal core-periphery organization predicts the rate of learning across individuals is compelling evidence that the relationship between geometrical and temporal core-periphery organization is related to learning. Nevertheless, it is important to ask whether changes in dynamic community structure and associated mesoscale network organization are related to tasks or to changes in subjects' physiological state over the course of longitudinal imaging [18]. It is clear from studies of behavior, peripheral physiology, and fMRI that subjects can have high levels of anxiety or stress (particularly during their first exposure to MRI) [19]. To address this issue, we describe additional evidence that supports our conclusions that the reported changes in dynamic community structure with learning are indeed related to motor tasks.

First, we note that we observed temporal and geometrical core-periphery organization consistently over all 4 scanning sessions. In Fig. S2 of the present document, we show that the anatomical identity of nodes in the temporal core, bulk, and periphery are consistent over scanning sessions. In Fig. S3 of this document, we show that the anatomical identity of nodes in the geometrical core and periphery are also consistent over scanning sessions. Moreover, Fig. 6 in the main manuscript shows that we observe the relationship between temporal and geometrical core-periphery organization consistently across scanning sessions.

Second, we assume that the effects of a subject's mental and physiological state (e.g., anxiety) are greatest during the first imaging session [20]. If this is indeed the case, then there could be significant changes of network organization between scans 1 (higher anxiety) and 2 (lower anxiety) that might lead to a spurious interpretation of changes in core-periphery organization. To examine this possibility, we test whether the changes in dynamic community structure and core-periphery organization with learning are robust to the removal of scan 1. Importantly, the trends in Figs. 2 and 5 in the main manuscript remain present if we only examine scans 2–4. We use data from scan 1 for the three box plots located at the point in the horizontal axis at which the number of trials is equal to 50. (This is the leftmost point of each panel.) See Table 1 in the main manuscript. The 9 box plots located at points on the horizontal axis at which the number of trials is greater than 50 use data from scans 2–4. Therefore, when we examine only scans 2–4, we still observe a decrease in maximum modularity, an increase in the number of communities, an increase in flexibility, and a decrease in the variance of the geometrical core score with learning.

Finally, task-related fMRI BOLD activation magnitude in core, bulk, and peripheral brain regions are not altered significantly across scanning sessions. We employed a repeated-measures analysis of variance (ANOVA) on the training-depth-averaged GLM parameter estimates [21]. We treated core, bulk, and periphery designations as categorical factors, and we treated scanning session as a repeated measure. We found a significant main effect (i.e., single-factor effect) of core, bulk, and periphery (an F-statistic [21] of $F(2, 38) \doteq 7.88$ and a p-value of $p \doteq 0.00137$) and a non-significant effect of scanning session ($F(3, 57) \doteq 0.615$, $p \doteq 0.584$). These results suggest that a systematic change in the hemodynamic response function across scanning sessions is unlikely to be responsible for the observed learning-related changes in dynamic community structure.

Furthermore, we observe that mean GLM parameter estimates in core, bulk, and peripheral brain regions are not correlated significantly with the reported changes in core-periphery structure that accompany learning. The Pearson correlation coefficient between parameter estimates and the variance of the geometrical core score for nodes in the temporal core is $r \doteq 0.20$ (which gives a p-value of $p \doteq 0.52$), for nodes in the temporal bulk is $r \doteq -0.05$ (so $p \doteq 0.86$), and for nodes in the temporal periphery is $r \doteq -0.52$ (so $p \doteq 0.08$). These results provide further evidence that BOLD activation magnitude and dynamic community structure provide distinct insights.

Temporal Core-Periphery Organization and Task-Related Activations

One of the strengths of our approach is that we examine the organization of whole-brain functional connectivity and thereby remain sensitive to a wide variety of learning-related changes in the brain that could not

be identified using a traditional GLM analysis. Nevertheless, it is useful to explore the relationship between dynamic community structure and task-related activations. In Fig. S6, we show that regions in the temporal core tend to be regions with strong task-related activations, as evinced by high (and positive) values of mean GLM parameter estimates. Conversely, regions in the temporal bulk and periphery tend to lack strong task-related activations, as evinced by low (and negative) values of mean GLM parameter estimates. These results are consistent with our interpretation that the temporal core consists of a small set of regions that are required to perform a given task and that the temporal periphery consists of a set of regions that are associated more peripherally with the task and which are activated in a transient manner.

Dynamic Community Detection

In the multilayer modularity quality function (see the "Materials and Methods" section of the main manuscript), we need to choose values for two parameters [6]: a structural resolution parameter γ and a temporal resolution parameter ω. We now examine the effects of these choices on our results.

Effect of Structural Resolution Parameter

In the main manuscript, we used a structural resolution parameter value of $\gamma = 1$, which is the most common choice when optimizing the single-layer and multilayer modularity quality functions [4, 5, 22]. In this case, $\mathbf{A} - \gamma\mathbf{P} = \mathbf{A} - \mathbf{P}$, and one is simply subtracting the optimization null model $\mathbf{P}$ from the adjacency tensor $\mathbf{A}$. One can decrease γ to access community structure at smaller spatial scales (i.e., to examine smaller communities) or increase it to access community structure at larger spatial scales (i.e., to examine larger communities). By examining network diagnostics over a range of γ values, we explore the spatial specificity of our results.

The mean number of communities in the partitions that we obtained by optimizing multilayer modularity Q varies from the minimum (1) to the maximum (112) possible value for γ approximately in the interval $[0.8, 2.5]$ (see Fig. 7A). We investigate this transition in greater detail in Figs. 7C,D. Near the value $\gamma = 1$, the number of regions in the bulk dips to about 65, whereas the number of regions in the core and periphery rise to about 20 and 25, respectively. Observe the dip of the bulk curve and bumps of the core and periphery curves in Fig. 7D. These features occur for γ approximately in the interval $[0.88, 1.22]$, which corresponds to partitions that are composed of between approximately 3 and approximately 20 communities (with an associated mean community size of between approximately 6 and approximately 37 brain regions; see Fig. 7B). This supports our claim that the temporal core-periphery structure that we examine in this study is a genuine mesoscale feature of coherent brain dynamics.

Effect of Temporal Resolution Parameter

In the main manuscript, we used a temporal resolution parameter value of $\omega = 1$. The value $\omega = 1$ ensures that the *inter*-layer coupling is equal to the maximum possible value of the *intra*-layer coupling, which we compute from the magnitude-squared coherence (which is constrained to lie in the interval $[0, 1]$). It is important to examine the robustness of results for different values of this parameter, and investigating dynamic network structure at other values of ω can also provide additional insights [6]. For example, one can decrease ω to encourage greater variability in community assignments of nodes across individual layers (i.e., across time in temporal networks) or increase it to encourage such community assignments to be more similar across layers. Recall that each node in the temporal multilayer network represents a single brain region at a specified time, and different nodes that represent the same brain region at different times become more likely to be assigned to the same multilayer community as ω is increased. By examining network diagnostics over a range of ω values, we can quantify the robustness of our results to differing amounts of temporal variation in community structure.

We varied ω from 0.1 to 2 in increments of $\Delta\omega = 0.1$. As expected, we find that the number of communities identified in the optimization of the multilayer modularity quality function decreases as ω is increased (see Fig. 8A). This is consistent with the fact that greater variation of community assignments across time is possible for smaller values of ω. Variation between community assignments of nodes in individual layers can occur in two ways: (1) a small number of regions change community membership from one layer to the next, but the majority of regions retain their community membership; or (2) entire communities lose their

identities (via fragmentation, extinction, union, and/or recombination), such that the algorithm identifies either the “death” of a community that was present in the previous layer but is not present in the current layer or the “birth” of a community that was not present in the previous layer but is present in the current layer.

For each value of ω, we examined the robustness of our division of brain regions into a temporal core, a temporal bulk, and a temporal periphery using the same procedure that we employed for $\omega = 1$. Namely, we defined a temporal core and temporal periphery as those brain regions that were composed, respectively, of the brain regions below and above the 95% confidence interval of the nodal null model. In Fig. 8B, we report the number of regions in each group as a function of ω. Interestingly, the number of brain regions that we identified as part of the temporal core varied little over the examined range of ω values; it remained at approximately 17.0 ± 1.1. In fact, 15 of the 17 regions that we identified as part of the temporal core at $\omega = 1$ were also identified as part of the temporal core at all other values of ω that we examined. The number of regions in the temporal bulk and temporal periphery varied more (with values of approximately 75.6 ± 7.4 for the bulk and approximately 19.4 ± 6.8 for the periphery), which suggests that the separation between the temporal bulk and temporal periphery is less drastic than that between temporal core and temporal bulk. Indeed, the mean flexibility of the core is less similar to the mean flexibility of the bulk than is the latter to the mean flexibility of the periphery. See Fig. 3 of the main manuscript and Figs. S1 and S2 of this supplement.

Figure S1. **Reliability of Temporal Core-Periphery Structure.** Temporal core (cyan), bulk (gold), and periphery (maroon) of dynamic networks determined based on the flexibility of trial blocks in which participants practiced sequences that would eventually be extensively trained. *(A)* Flexibility of the temporal core, bulk, and periphery averaged over the 100 multilayer modularity optimizations and 20 participants for blocks composed of extensively trained (EXT; light circles), moderately trained (MOD; squares), and minimally trained (MIN; dark diamonds) sequences. The darkness of data points indicates scanning session; darker colors indicate earlier scans, so the darkest colors indicate scan 1 and the lightest ones indicate scan 4. *(B)* The coefficient of variation of flexibility calculated over the 100 optimizations and 3 sequence types for all brain regions. Error bars indicate the standard error of the mean CV over participants. Both panels use data from scanning session 1 on day 1 of the experiment (which is prior to home training).

Figure S2. **Temporal Core-Periphery Organization Over 42 Days.** Temporal core (cyan), bulk (gold), and periphery (maroon) of dynamic networks defined by trial blocks in which participants practiced sequences that would eventually be *(A)* extensively trained, *(B)* moderately trained, and *(C)* minimally trained for data from scanning sessions 2 (after approximately 2 weeks of training; circles), 3 (after approximately 4 weeks of training; squares), and 4 (after approximately 6 weeks of training; diamonds). The darkness of data points indicates scanning session; darker colors indicate earlier scans, so the darkest colors indicate scan 1 and the lightest ones indicate scan 4.

Figure S3. **Geometrical Core-Periphery Organization Over 42 Days.** Geometrical core scores for each brain region defined by the trial blocks in which participants practiced sequences that would eventually be *(A)* extensively trained, *(B)* moderately trained, and *(C)* minimally trained for data from scanning sessions 1 (day 1; black circles), 2 (after approximately 2 weeks of training; dark gray squares), 3 (after approximately 4 weeks of training; gray diamonds), and 4 (after approximately 6 weeks of training; light gray stars). We have averaged the geometrical core scores over blocks and over 20 participants. The order of brain regions is identical for all 3 panels (*A*-*C*), and we chose this order by ranking regions from high to low geometrical core scores from the EXT blocks on scanning session 1 (on day 1 of the experiment).

Figure S4. **Relationship Between Temporal Core-Periphery Organization and Community Structure.** *(A)* Mean-coherence matrix over all EXT blocks from all participants on scanning day 1. The colored bars above the matrix indicate the 3 communities that we identified from the representative partition. Mean partition similarity z-score z_i over all participants for blocks of *(B)* extensively, *(C)* moderately, and *(D)* minimally trained sequences for all 4 scanning sessions over the approximately 6 weeks of training. The horizontal gray lines in panels *(B-D)* indicate the z_i value that corresponds to a right-tailed p-value of 0.05.

Figure S5. **Region Size is Uncorrelated with Flexibility.** *(A)* Scatter plot of the size of the brain region in voxels (averaged over participants) versus the flexibility of the EXT multilayer networks, which we averaged over the 100 multilayer modularity optimizations and the 20 participants. Data points indicate brain regions. The line indicates the best linear fit. Its Pearson correlation coefficient is $r \doteq -0.009$, and the associated p-value is $p \doteq 0.92$. *(B)* Box plot over the 20 participants of the squared Pearson correlation coefficient r^2 between the participant-specific region size in voxels and the participant-specific flexibility averaged over the 100 multilayer modularity optimizations.

Figure S6. **Temporal Core-Periphery Organization and Task-Related Activations.** Mean GLM parameter estimates for the temporal core (cyan; circles), bulk (gold; squares), and periphery (maroon; diamonds) of dynamic networks defined by the trial blocks in which participants practiced sequences that would eventually be *(A)* extensively trained, *(B)* moderately trained, and *(C)* minimally trained for data from scanning sessions 1 (first day of training), 2 (after approximately 2 weeks of training), 3 (after approximately 4 weeks of training), and 4 (after approximately 6 weeks of training).

Figure S7. **Effect of Structural Resolution Parameter.** *(A,B)* Number of communities and *(C,D)*

number of regions in the temporal core (cyan; circles), temporal bulk (gold; squares), and temporal periphery (maroon; diamonds) as a function of the structural resolution parameter γ, where we considered *(A,C)* $\gamma \in [0.2, 5]$ in increments of $\Delta\gamma = 0.2$ and *(B,D)* $\gamma \in [0.8, 1.8]$ in increments of $\Delta\gamma = 0.01$. We averaged the values in panels *(A)* and *(B)* over 100 multilayer modularity optimizations and over the 20 participants.

Figure S8. **Effect of Temporal Resolution Parameter.** *(A)* Number of communities averaged over 100 multilayer modularity optimizations and over 20 participants as a function of the temporal resolution parameter ω. *(B)* Number of regions that we identified as part of the temporal core (cyan; circles), temporal bulk (gold; squares), and temporal periphery (maroon; diamonds) as we vary ω from 0.1 to 2 in increments of $\Delta\omega = 0.1$.

References

[1] Newman MEJ, Girvan M (2004) Finding and evaluating community structure in networks. Phys Rev E 69: 026113.

[2] Newman MEJ (2004) Fast algorithm for detecting community structure in networks. Phys Rev E 69: 066133.

[3] Newman MEJ (2006) Modularity and community structure in networks. Proc Natl Acad Sci USA 103: 8577–8582.

[4] Porter MA, Onnela JP, Mucha PJ (2009) Communities in networks. Not Amer Math Soc 56: 1082–1097, 1164–1166.

[5] Fortunato S (2010) Community detection in graphs. Phys Rep 486: 75–174.

[6] Bassett DS, Porter MA, Wymbs NF, Grafton ST, Carlson JM, et al. (2012) Robust detection of dynamic community structure in networks. Chaos 23: 013142.

[7] Simpson SL, Moussa MN, Laurienti PJ (2012) An exponential random graph modeling approach to creating group-based representative whole-brain connectivity networks. NeuroImage 60: 1117–1126.

[8] Traud AL, Kelsic ED, Mucha PJ, Porter MA (2011) Comparing community structure to characteristics in online collegiate social networks. SIAM Rev 53: 526–543.

[9] Hagmann P, Cammoun L, Gigandet X, Meuli R, Honey CJ, et al. (2008) Mapping the structural core of human cerebral cortex. PLoS Biol 6: e159.

[10] Bassett DS, Brown JA, Deshpande V, Carlson JM, Grafton ST (2011) Conserved and variable architecture of human white matter connectivity. NeuroImage 54: 1262–1279.

[11] MATLAB (2012) Measuring signal power. MATLAB's Help Menu .

[12] Smith SW (1997) Digital Signal Processing: A Guide for Engineers and Scientists. California Technical Pub.

[13] Lazar N (2010) The Statistical Analysis of Functional MRI Data. Springer-Verlag.

[14] Friston KJ, Holmes AP, Worsley KJ, Poline JP, Frith CD, et al. (1994) Statistical parametric maps in functional imaging: A general linear approach. Human Brain Mapping 2: 189–210.

[15] Grinband J, Wager TD, Lindquist M, Ferrera VP, Hirsch J (2008) Detection of time-varying signals in event-related fMRI designs. NeuroImage 43: 509–520.

[16] Steffener J, Tabert M, Reuben A, Stern Y (2010) Investigating hemodynamic response variability at the group level using basis functions. NeuroImage 49: 2113–2122.

[17] Calhoun VD, Adali T, Pekar JJ (2004) A method for comparing group fMRI data using independent component analysis: application to visual, motor and visuomotor tasks. Magn Reson Imaging 22: 1181–1191.

[18] Johnstone T, Somerville LH, Alexander AL, Oakes TR, Davidson RJ, et al. (2005) Stability of amygdala BOLD response to fearful faces over multiple scan sessions. NeuroImage 25: 1112–1123.

[19] Lueken U, Muehlhan M, Evens R, Wittchen HU, Kirschbaum C (2012) Within and between session changes in subjective and neuroendocrine stress parameters during magnetic resonance imaging: A controlled scanner training study. Psychoneuroendocrinology 37: 1299–1308.

[20] Chapman HA, Bernier D, Rusak B (2010) MRI-related anxiety levels change within and between repeated scanning sessions. Psychiatry Res 182: 160–164.

[21] Agresti A, Franklin CA (2007) Statistics: The Art and Science of Learning From Data. Prentice Hall.

[22] Reichardt J, Bornholdt S (2006) Statistical mechanics of community detection. Phys Rev E 74: 016110.
